# Right-sided minimally invasive direct coronary artery bypass: Preoperative planning and surgical technique

**DOI:** 10.1016/j.xjtc.2024.02.015

**Published:** 2024-02-29

**Authors:** Florian Hecker, Razan Salem, Mascha von Zeppelin, Jan Hlavicka, Thomas Walther, Tomas Holubec

**Affiliations:** Department of Cardiovascular Surgery, University Hospital Frankfurt and Goethe University Frankfurt, Frankfurt, Germany

**Figure:**
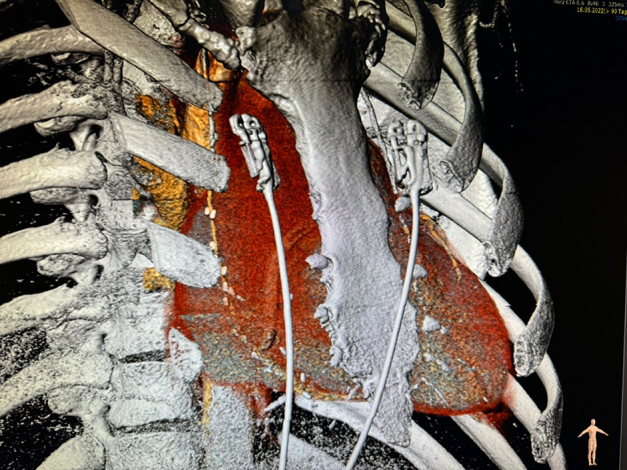
We present our approach to the r-MIDCAB technique.

**Central Message**

Right-sided minimally invasive direct coronary artery bypass (r-MIDCAB) can offer an attractive minimally invasive alternative to full sternotomy in isolated complex right coronary artery (RCA) disease or in anomalous RCA.

Left-sided minimally invasive direct coronary artery bypass (MIDCAB) has gained widespread popularity since its introduction during the 1990s for the treatment of isolated left anterior descending artery stenosis or as part of a hybrid approach in multivessel coronary artery disease.^1^^,^^2^ In isolated complex right coronary artery (RCA) disease or in anomalous RCA, right-sided minimally invasive coronary artery bypass (r-MIDCAB) can offer an attractive minimally invasive alternative to full sternotomy.^3^ We aim to present our approach to r-MIDCAB using right internal thoracic artery (RITA) to RCA, based on our experience with nearly 20 cases.

Informed consent for the publication of the study data was obtained from patient and no institutional review board approval is needed for this report according to German law.

## Preoperative Assessment

In addition to the routine preoperative diagnostic workup, consisting of coronary angiogram, transthoracic echocardiography, and chest radiograph, we perform a computed tomography scan and analyze it using 3mensio Structural Heart software (3mensio Medical Imaging), which is crucial to determine the optimal intercostal space (ICS) and accessibility of RCA. Zone 3 is generally the most distal part of the RCA for comfortable anastomosis. Sometimes posterior descending artery can be reached and anastomosed; however, there is no guarantee, so we tend to refrain from r-MIDCAB in these cases.

## Surgical Technique

**Figure 1:**
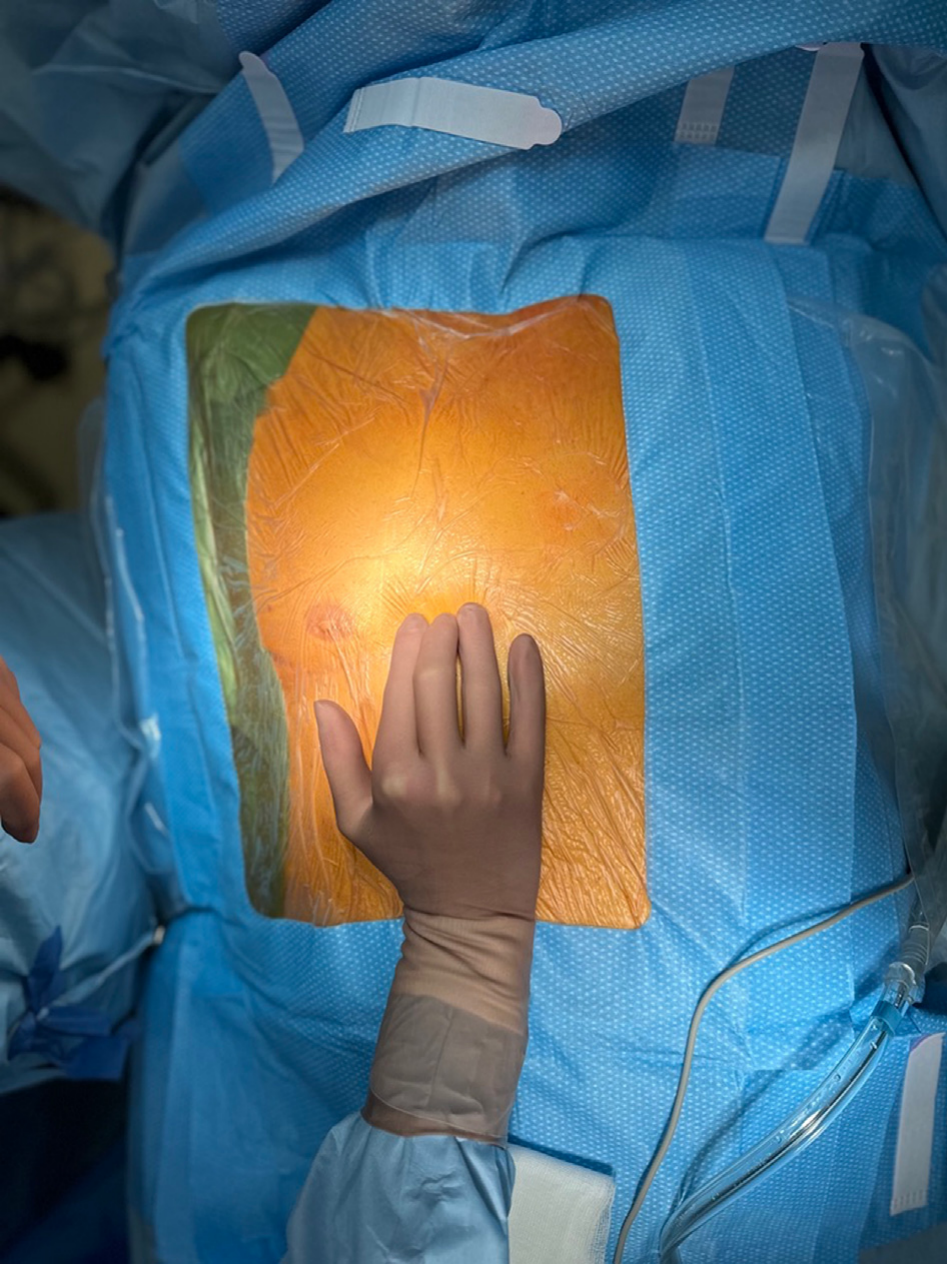
Intraoperative image showing palpation of the fifth intercostal space.

As in left-sided MIDCAB, the use of unilateral lung ventilation is employed in all patients through either double-lumen tracheal intubation or selective endoluminal blockage guided by bronchoscopy. Transesophageal echocardiography is standard across all cases. The patient is put in the supine position, with a 30° elevation of the chest on the right side facilitated by the placement of a soft pillow beneath the scapula (Figure 1).

**Figure 2:**
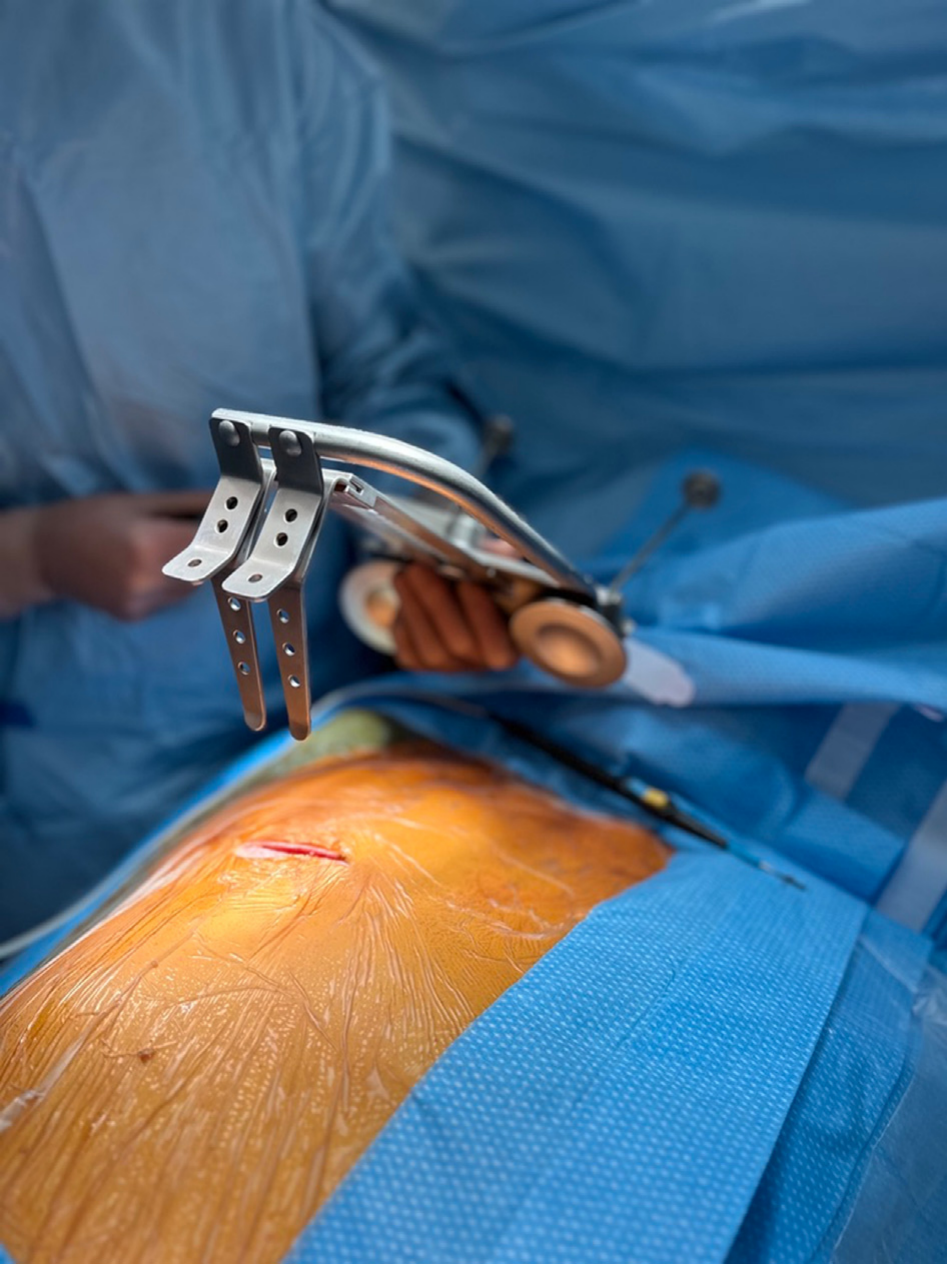
Intraoperative picture of the surgical setting for preparation of the right internal thoracic artery after minithoracotomy at the fifth intercostal space before inserting the rib lifting retractor.

**Figure 3:**
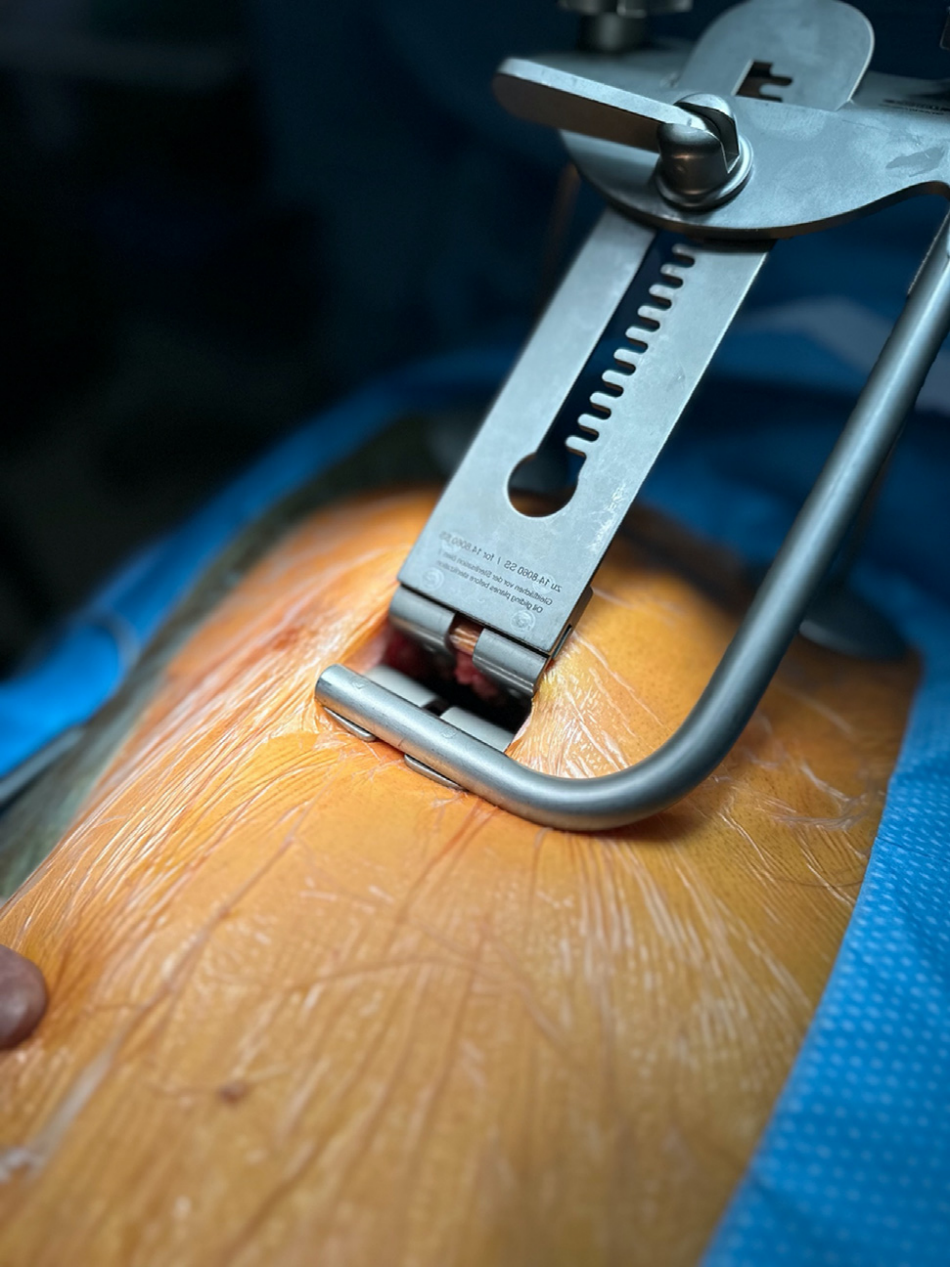
Intraoperative picture of the surgical setting for preparation of the right internal thoracic artery after placement of the rib lifting retractor in the fifth intercostal space.

The surgical procedure is carried out through a 5- to 6-cm long right anterior minithoracotomy (Figure 2). The RITA is harvested under direct vision using a Hauser retractor (B.Braun/Aesculap AG) in a skeletonized technique using a regular diathermal blade and hemostatic clips. It is paramount to retract the ribs gradually to avoid rib fractures (Figure 3). Unlike the left-sided MIDCAB, where the fourth ICS provides optimal access for left ITA harvesting almost in all cases, in the majority of r-MIDCAB, the fifth ICS is utilized to maximize the length of the RITA. The length of the harvested RITA corresponds nearly to the length achieved through a median sternotomy, sometimes even longer. Proximal harvesting of the RITA extends up to the first ICS, close to the subclavian vein, whereas distal harvesting reaches until the bifurcation. Systemic heparin (1 mg/kg) is administered to achieve an activated clotting time of at least 300 seconds during the surgery. Subsequently, the RITA is divided, and the distal end is secured to the skin level using a 6–0 polypropylene suture.

The pericardium is then opened ventrally over the right atrium, which is retracted by pericardial stay sutures for ventral luxation of the heart. A reusable mechanical MIDCAB stabilizer (Fehling Instruments) facilitates stabilization of the targeted area for anastomosis to the RCA. Proximal snaring of the RCA is achieved using a silastic hollow vessel loop with a blunt-tipped needle (Retract-O-Tape; Quest Medical, Inc.). The anastomosis is executed in a standard end-to-side off-pump fashion using 8–0 polypropylene sutures, with the routine incorporation of a coronary shunt (Clearview; Medtronic), and a carbon dioxide blower (Accumist Blower/Mister; Medtronic) for optimal visualization of the operative field (Video 1).

Following the completion of the anastomosis, the patency of the bypass is confirmed through transit time flow measurement using the MiraQ Cardiac system (Medistim ASA). The pericardium is closed, allowing unobstructed course of RITA into the pericardium through attaching the fatty pad margin to the medial portion of the pericardium at the basal portion of the pericardiotomy. It is important to cover the anastomosis site to protect it from lung rubbing, which can cause kinking or rotation. A 28 F chest drain is inserted into the right pleural cavity through the sixth ICS. The minithoracotomy closure is accomplished using a resorbable polyglyactin 2–0 Z-suture of the ribs, followed by layered wound closure. Postoperative pain management is addressed through the administration of periosteal and intramuscular 0.7% bupivacaine.

## Postoperative Course

The majority of patients undergo extubation in the operating room. They are promptly transitioned to the intensive or intermediate care unit for further monitoring and care. Initiation of postoperative pharmacotherapy includes the administration of acetylsalicylic acid at a daily dose of 100 mg on the first postoperative day (lifelong), and additionally a P2Y12-receptor antagonist on the second postoperative day (clopidogrel or ticagrelor) for 6 months postoperatively.

## Discussion

Right-sided MIDCAB has proofed its feasibility and safety in isolated complex RCA disease or in anomalous RCA, as published in our initial experience with 11 cases.^3^ In the meantime, we have successfully operated on another 7 patients. In our opinion, minimally invasive RITA harvesting and its anastomosis to RCA in zone 2 is similarly demanding as in regular left-sided MIDCAB. More distal anastomosis may be more challenging and requires sometimes a diaphragmic traction suture in the tendinous center for luxation of the heart and better exposure. The patency of the RITA graft remains excellent with up to 90% at 10 years based on angiographic studies.^4^ Therefore, r-MIDCAB offers an attractive minimally invasive alternative for selected patients with single-vessel disease with respect to the location of the stenosis.

## Conflict of Interest Statement

The authors reported no conflicts of interest.

The *Journal* policy requires editors and reviewers to disclose conflicts of interest and to decline handling manuscripts for which they may have a conflict of interest. The editors and reviewers of this article have no conflicts of interest.

## Supplementary Data

Video 1 Intraoperative video showing harvested right internal thoracic artery (RITA) and execution of the anastomosis of RITA to distal right coronary artery in a standard end-to-side off-pump fashion. Video available at: https://www.jtcvs.org/article/S2666-2507(24)00072-5/fulltext.
