# Supplementary figures and images for: Right-sided minimally invasive direct coronary artery bypass: Preoperative planning and surgical technique
Source: JTCVS Tech. 2024 Feb 29;25:94–6. doi: 10.1016/j.xjtc.2024.02.015 (PMC11184483; doi:10.1016/j.xjtc.2024.02.015)

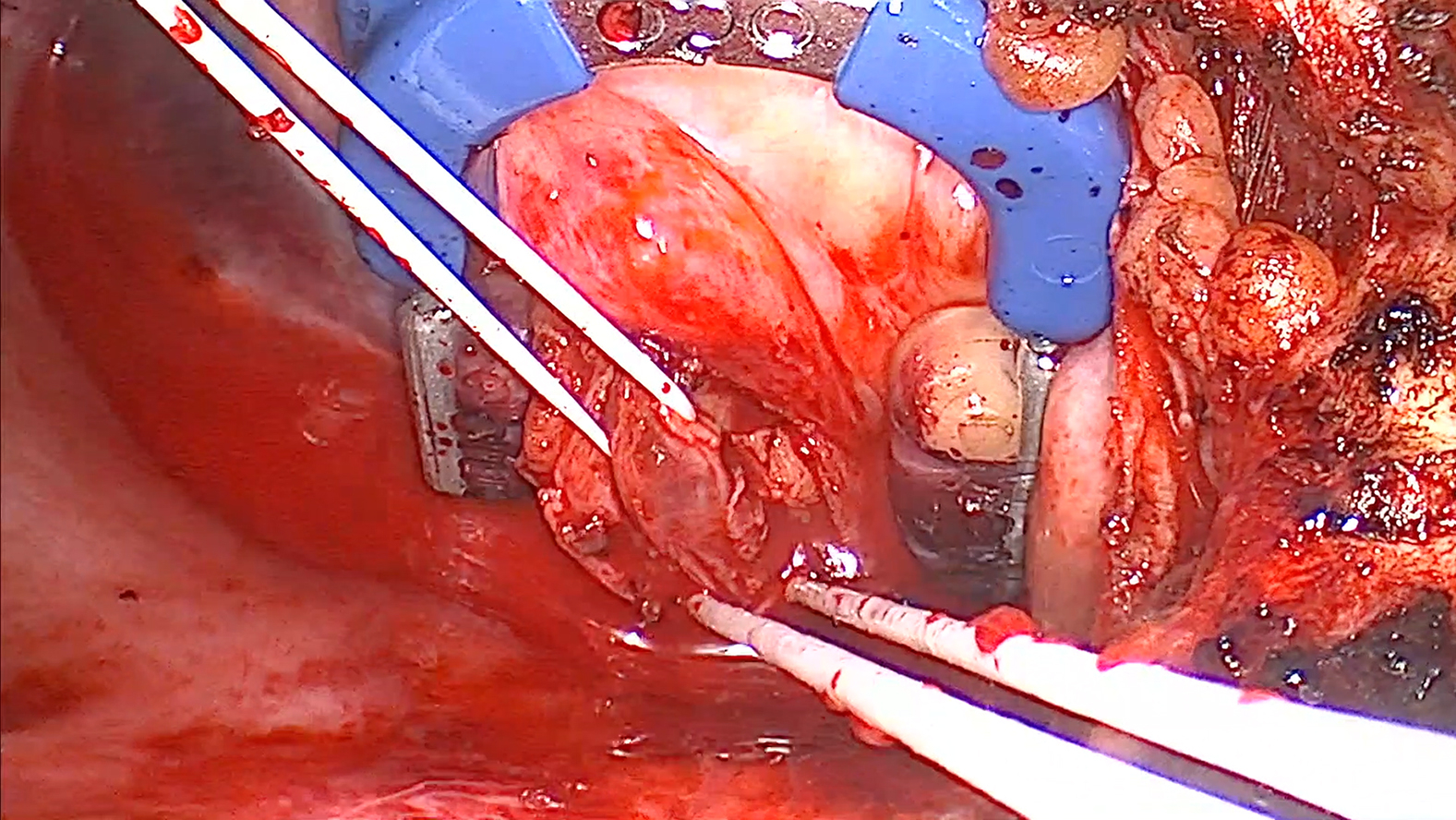

Supplement: Video 1 — Intraoperative video showing harvested right internal thoracic artery (RITA) and execution of the anastomosis of RITA to distal right coronary artery in a standard end-to-side off-pump fashion. Video available at: https://www.jtcvs.org/article/S2666-2507(24)00072-5/fulltext. [file fx2.jpg]
